# Genome-wide DNA methylation at birth in relation to in utero arsenic exposure and the associated health in later life

**DOI:** 10.1186/s12940-017-0262-0

**Published:** 2017-05-30

**Authors:** Akhilesh Kaushal, Hongmei Zhang, Wilfried J. J. Karmaus, Todd M. Everson, Carmen J. Marsit, Margaret R. Karagas, Shih-Fen Tsai, Hui-Ju Wen, Shu-Li Wang

**Affiliations:** 1 Division of Epidemiology, Biostatistics, and Environmental Health, University of Memphis, Memphis, TN 38152 USA; 2 Department of Environmental Health, Rollins School of Public Health, Emory University, Atlanta, GA USA; 3 Department of Epidemiology, Geisel School of Medicine at Dartmouth College, Hanover, NH USA; 4 Children’s Environmental Health & Disease Prevention Research Center at Dartmouth, Hanover, NH USA; 5 National Institute of Environmental Health Sciences, National Health Research Institutes, Miaoli, Taiwan; 6 School of Public Health, National Defense Medical Center, Taipei, Taiwan; 7 Department of Public Health, China Medical University, Taichung, Taiwan; 8 Research Center for Environmental Medicine, Kaohsiung Medical University, Kaohsiung, Taiwan

**Keywords:** Arsenic, DNA methylation, CpG, DAVID, KEGG pathway, Genome-wide, Ldl

## Abstract

**Background:**

In utero arsenic exposure may alter fetal developmental programming by altering DNA methylation, which may result in a higher risk of disease in later life. We evaluated the association between in utero arsenic exposure and DNA methylation (DNAm) in cord blood and its influence in later life.

**Methods:**

Genome-wide DNA methylation in cord blood from 64 subjects in the Taiwanese maternal infant and birth cohort was analyzed. Robust regressions were applied to assess the association of DNA methylation with in utero arsenic exposure. Multiple testing was adjusted by controlling false discovery rate (FDR) of 0.05. The DAVID bioinformatics tool was implemented for functional annotation analyses on the detected CpGs. The identified CpGs were further tested in an independent cohort. For the CpGs replicated in the independent cohort, linear mixed models were applied to assess the association of DNA methylation with low-density lipoprotein (LDL) at different ages (2, 5, 8, 11 and 14 years).

**Results:**

In total, 579 out of 385,183 CpGs were identified after adjusting for multiple testing (FDR = 0.05), of which ~60% were positively associated with arsenic exposure. Functional annotation analysis on these CpGs detected 17 KEGG pathways (FDR = 0.05) including pathways for cardiovascular diseases (CVD) and diabetes mellitus. In the independent cohort, about 46% (252 out of 553 CpGs) of the identified CpGs showed associations consistent with those in the study cohort. In total, 11 CpGs replicated in the independent cohort were in the pathways related to CVD and diabetes mellitus. Via longitudinal analyses, we found at 5 out of the 11 CpGs methylation was associated with LDL over time and interactions between DNA methylation and time were observed at 4 of the 5 CpGs, cg25189764 (coeff = 0.157, *p*-value = 0.047), cg04986899 (coeff. For interaction [coeff.int] = 0.030, *p*-value = 0.024), cg04903360 (coeff.int = 0.026, *p*-value = 0.032), cg08198265 (coeff.int = −0.063, *p*-value = 0.0021), cg10473311 (coeff.int = −0.021, *p*-value = 0.027).

**Conclusion:**

In utero arsenic exposure was associated with cord blood DNA methylation at various CpGs. The identified CpGs may help determine pathological epigenetic mechanisms linked to in utero arsenic exposure. Five CpGs (cg25189764, cg04986899, cg04903360, cg08198265 and cg10473311) may serve as epigenetic markers for changes in LDL later in life.

**Electronic supplementary material:**

The online version of this article (doi:10.1186/s12940-017-0262-0) contains supplementary material, which is available to authorized users.

## Background

Arsenic, a widespread element in the environment, poses a serious threat to human health. Millions of people around the globe are exposed to arsenic from drinking water that exceeds the safe limit of 10 ppb as recommended by World Health Organizations [1]. Arsenic is known to easily pass through the placenta in humans and other mammals, producing arsenic concentrations in cord blood similar to maternal blood [2]. Epidemiological studies have reported that gestational arsenic exposure is associated with increased risk of non-cancerous and cancerous diseases in adulthood [3, 4]. For instance, a number of studies have shown that early life arsenic exposure is associated with later cardiovascular diseases (CVDs) [5–7]. In animal studies, in utero exposure to low level arsenic in the womb and in adulthood was found to be associated with diabetes mellitus [8].

The mechanisms through which in utero exposure to arsenic may result in a higher risk of various diseases are not well understood. However, harmful effects such as the generation of reactive oxygen species (ROS), which causes oxidative DNA damage, binding and inhibition of arsenic metabolites to enzymes, and perturbation of key signaling pathways, are thought to play certain roles in disease development [9]. In addition, clinical and epidemiological studies have observed that environmental exposure in early life can affect the risk of disease later in life through a phenomenon known as developmental programming [4, 10, 11]. The study of epigenetic changes such as DNA methylation alterations that can affect gene activity may provide insight into developmental programming [12].

Studies found that chronic arsenic exposure in adults is associated with increased DNA methylation extracted from whole blood leukocytes [13, 14]. Experimental studies in animals have also shown that intra-uterine exposure to arsenic alters DNA methylation in offspring [15]. Some studies examined the association of genome-wide DNA methylation in cord blood with in utero arsenic exposure [16–18]. These studies were based on cohorts established in the United States [16], Mexico [17] and Bangladesh [18]. Some of the studies did not identify any statistically significant CpGs at the whole epigenome level, and thus focused on the top 100 [16] or 500 CpG sites [18] potentially associated with in utero arsenic exposure. The study by Kile et al. [19] investigated the association of CpG sites in *p16, p53*, LINE-1 and Alu repetitive elements. Rojas et al. [17], on the other hand, did identify a set of statistically significant CpGs associated with in utero arsenic exposure in a cohort established in Mexico.

Our study, based on data from a prospective birth cohort study established in Taiwan, aimed to comprehensively assess genome-wide DNA methylation in cord blood in association with in utero arsenic exposures (using maternal urinary arsenic concentrations), identify CpG sites showing such statistically significant associations after adjusting for multiple testing by controlling false discovery rate (FDR), and examine possible pathways of genes involving the identified CpGs. Additionally, we attempted to replicate our finding in an independent birth cohort (New Hampshire birth cohort study; NHBCS) and further assessed longitudinal associations of DNA methylation with disease biomarkers measured at later ages in our cohort from Taiwan. The findings will contribute to an improved understanding of the adverse mechanisms of in utero arsenic exposure on genome-wide epigenetic variation and whether epigenetic markers in cord blood can influence children’s diseases risk later in life.

## Methods

### Taiwanese maternal infant and Birth Cohort description

The data resulted from the Maternal and Infant Cohort Study in Taiwan investigating various in utero and postnatal factors considered to affect child health outcomes [4]. All pregnant women participating in this study signed informed consent forms explaining the benefits and risks of participation. This study was approved by Human Ethical Committee of the National Health Research Institutes in Taiwan. Pregnant women who received medical care at a local medical center were invited to join this study between December 2000 and November 2001. Among the 610 women who met the requirement, 430 volunteered to participate in the study (the flow of data collection in Additional file 1: Figure S1). Of the 430 pregnant women, 117 were excluded due to non-compliance of providing samples. Urine samples were then collected from the remaining 313 pregnant women during the third trimester (28–38 weeks of gestation). In total 313 livebirths were reported as noted in our earlier work [4]. Out of the 313 livebirths 9 were twins and one of the twins was randomly selected for subsequent studies. In addition, five newborns could not be included due to loss of follow up. This resulted in 299 mother-newborn pairs. The cord blood sample was collected for all the 299 mother-newborn pairs. DNA methylation was measured for 64 cord blood samples that had required DNA concentration and quality for this epigenome assay.

Data Collection, Pre-processing, and Cell Mixture Assessment (Additional file 2: Material 1).

Participants provided a spot urine sample at the time of enrollment in this study (at 28–38 weeks of gestation), and Arsenite (As^III^), arsenate (As^V^), monomethylarsonic acid (MMA), and dimethylarsinic acid (DMA) were quantified using high-performance liquid chromatography/inductively coupled plasma mass spectrometry (HPLC-ICP-MS). Anion exchange columns were used (Hamilton PRP X-100 [10 μm particle size, 250 mm × 4.1 mm]) for arsenic speciation. Creatinine was measured by the Beckman Synchron LX20 auto-system (Beckman Coulter, Brea, CA, USA) in the central lab of Chung-Ho Memorial Hospital of Kaohsiung Medical University using a spectrophotometric method with picric acid as the reactive at 520 nm.

DNA was isolated from cord blood samples and DNA methylation was measured using Illumina Infinium HumanMethylation 450 BeadChip (Illumina, San Diego, CA). DNA methylation was pre-processed using the Bioconductor *minfi* package. Cell type proportions of six cells were estimated using the R function e*stimateCellCounts* in the R package *minfi* [20, 21]. Detailed information of this section can be found in Additional file 2: Material 1. The LDL Cholesterol Direct method was used to measure LDL cholesterol from the serum and plasma of the participants using the ADVIA Chemistry systems.

### Replication study

The replication study was conducted within the New Hampshire Birth Cohort Study (NHBCS) described elsewhere [22]. Details about the replication sample are provided in the supplemental materials (Additional file 3: Material 2). Briefly, the NHBCS began enrollment in 2009 and is an ongoing prospective birth cohort in the northeastern United States aimed at studying environmental and lifestyle factors that may impact the health of pregnant mothers and their children. Spot urine samples were collected between 24 and 28 weeks gestation. DNA methylation from cord blood was assessed using the Illumina Infinium HumanMethylation450 BeadChip.

### Statistical analyses

The dataset consists of 64 samples from cord blood specimen with DNA methylation data for 485,577 CpG site. Preprocessing of DNAm was performed using Subset-quantile Within Array Normalization (SWAN) [23] available in Bioconductor package minfi [24]. The preprocessing deleted 65 control probe CpG sites, 16,632 CpG sites with detection *p*-value > 0.01, 11,648 CpG sites that were located on X or Y chromosomes, and 72,049 located on probe SNPs or were within 10 base pairs of the probe SNPs. After the quality control, 385,183 CpG sites were retained for statistical analysis. The pre-processed DNAm data in beta values were transformed to M values, approximated as log2 [β/(1-β)], in order to ensure a better fit to statistical model assumptions used in our analyses.

To identify CpG sites whose DNAm is influenced by in utero arsenic exposure (tAs), robust regressions (lmFit function R-package *limma*) [25] were applied to model the association of DNAm with urinary creatinine-adjusted total arsenic (tAs). Child’s sex, batch effect, mother’s age, mothers pre-pregnancy BMI, and education level, and estimated blood cell proportions (CD8T, CD4T, NK, and B-cells, monocytes and granulocytes [20, 21]) were included as covariates. Robust regressions in *limma* package use an empirical Bayes approach to estimate sample variances which provides stable inference when the number of arrays is small [26]. In the robust regression analyses, multiple testing is adjusted by controlling FDR of 0.05. For the replication analyses we reproduced the statistical models described above in the NHBCS sample. CpGs with regression coefficients are in the same directions were considered to be successfully replicated, and we attempted to control for multiple testing via FDR of 0.05.

To assess the association of DNA methylation at CpGs of genes in some of the identified pathways with longitudinal (2, 5, 8, 11 and 14 years) low-density lipoprotein (LDL), a biomarker for CVD and diabetes, we applied linear mixed models. Log_10_ LDL concentrations at different ages were the dependent variable and residuals of DNA methylation, age, as well as interaction between age and DNA methylation were included in the model as predictors, and sex, birth weight, were treated as covariates. Since BMI is known to be associated with LDL among children [27], to assess potential confounding effects, we performed another analysis by including children’s BMI Z-scores at the ages of 2, 5, 8, 11, and 14 years into the linear mixed model. BMI was calculated as weight (kg) divided by height squared (m^2^). A BMI Z-Score of a subject was calculated as the ratio of difference between the subject’s BMI and BMI sample mean over the sample standard deviation of BMI. To further assess possible mediation effects of DNA methylation on the connection between arsenic exposure and LDL, we evaluated the association of in utero arsenic exposure with LDL at different ages (2, 5, 8, 11, and 14 years) using a linear mixed model. A statistical significance level was set at 0.05. The residuals of DNA methylation were obtained by regressing DNA methylation at each of 12 CpG sites on proportions of each of the six cell types (CD8T, CD4T, NK, and B-cells, monocytes and granulocytes) and batch.

### Pathway analyses (Additional file 2: Material 1)

Database for Annotation, Visualization and Integrated Discovery (DAVID) [28] was used to identify the enriched pathways associated with genes linked to the identified CpG sites. Detailed information on DAVID is in Additional file 2: Material 1.

### Accessible resource for integrated Epigenomic studies (ARIES) and Assessment of DNAm stability

ARIES is based on a sub-cohort of the Avon Longitudinal Study of Parents and Children (ALSPAC) [29, 30]. It provides population based resource of DNA methylation data. ARIES consists of 1018 mother-offspring pairs with DNA samples at two time points for the mother (at an antenatal clinic and at a follow-up clinic when their offspring around age 15 years) and three time points for the offspring (at birth, childhood around 7 years, and adolescence around 15 years). DNA methylation for children at birth was derived from cord blood, while at later ages it was from peripheral blood. Stability of DNA methylation for each CpG site was assessed by Gene view in ARIES explorer (http://www.ariesepigenomics.org.uk/ariesexplorer). This explorer lists all the CpG sites related to the specific genes. The stability of DNAm at a CpG site was assessed by comparing the median/variance of beta values at different ages of mothers as well as different ages of their offspring (birth, 7 years, and 15–17 years). CpG sites with approximately constant median/variance of beta values were considered stable.

## Results

The data were from a birth cohort study examining multiple in utero and postnatal factors in relation to child health outcomes as part of the nationwide Taiwan Maternal and Infant Cohort Study established in Taiwan in 2000–2001 [4]. In total, 64 subjects with genome-wide DNA methylation in cord blood, level of maternal urinary arsenic exposure, urinary creatinine, along with a child’s sex, gestational age, maternal age, maternal pre-pregnancy body mass index (BMI) and the mother’s educational level were available and utilized in the study. Table 1 presents a comparison of characteristics of 64 subjects in the study with those from whole cohort (*n* = 299). The pre-pregnancy BMI and education level in the study sample were likely to be different from those in the whole cohort (Table 1). Table 2 compares the characteristics of pregnant women and newborns by sex. Of the 64 newborns, 38 (59.4%) were male. Maternal characteristics are comparable between male and female newborns, and there is no statistically significant difference in gestational ages between sexes of newborns.

**Table 1:** Comparison of study sample with the whole cohort

	Study sample (*n* = 64)	All mothers and their newborn (*n* = 299)	
Variables	Mean ± SD. or number(%) or Median(IQR)	Mean ± SD. or number(%) or Median(IQR)	*p*-value
Maternal Characteristics			
Age (Years)	28.9 ± 4.8	28.3 ± 4.2	0.32
Pre-pregnant BMI (kg/m^2^)	20.5 ± 2.6	25.6 ± 3.9	1.92 × 10^−23^
Arsenic metabolites μg g^−1^ crea^a^			
Total arsenic	23.19(21.2)	22.26(28.51)	0.52
Inorganic arsenic	0.83(0.92)	0.79(1.11)	0.9
MMA	0.40(1.48)	0.46(1.85)	0.7
DMA	20.73(18.23)	20.01(24.82)	0.52
*Maternal Education*			5.12 × 10^−15^
≤high school	3 (4.7%)	132(44%)	
high school +2 years	22(34.3%)	117(39%)	
≥high school +4 years	39(61%)	50(17%)	
Newborns			
Gestational Age (weeks)	39 ± 1.2	39 ± 2.8	1

^a^: μg g^−1^ crea: μg per g creatinine

**Table 2:** Characteristics of mothers and their newborns by newborn sex in Taiwan during 2000–2001 (*n* = 64)

		Sex of the infant		
Characteristics	All (*n* = 64)^a^	Male (*n* = 38)^a^	Female (*n* = 26)^a^	*p*-value^b^
Pregnant Women				
Age (years)	28.9 ± 4.8	28.6 ± 4.1	29.5 ± 5.7	0.492
Pre-pregnant BMI (Kg/m2)	20.5 ± 2.6	20.2 ± 2.4	21.0 ± 2.9	0.244
Urinary Creatinine (mg/dL)	63.6 ± 41.7	70.9 ± 46.0	53.0 ± 32.9	0.078
Maternal Education				0.303
high school +2 years	25(39)	13(34)	12(48)	
≥high school +4 years	39(61)	25(66)	14(52)	
Newborns				
Gestational Age (weeks)	39 ± 1.2	39 ± 1.1	39 ± 1.4	0.791

^a^Presented as the mean ± SD or number (percentage).

^b^ *p*-value for difference between male and female newborns using t-test for continuous variables and χ^2^ or Fishers Exact Test for categorical variable

The levels and distribution of arsenic metabolites in maternal urine after adjusting for creatinine levels are shown in Table 3, distinguishing between mono-methylated arsenic (MMA), di-methylated arsenic (DMA), inorganic arsenic (iAs), and the sum of the three (total arsenic or tAs). Concentrations of each urinary arsenic species showed a large variation among the 64 mothers. We focused on tAs to represent overall arsenic exposure. The distribution of tAs is severely skewed with a median of 23.19 μg per gram creatinine (μg g^−1^ crea [creatinine]), and 5th and 95th percentiles being 3.76 μg g^−1^ crea and 76.02 μg g^−1^ crea, respectively (Table 3 and Additional file 4: Figure S2). The results reported in this article are based on log10-transformed total arsenic concentration.

**Table 3:** Distribution of creatinine-adjusted concentrations of urinary arsenic species (iAs, MMA, and DMA) (*n* = 64)

Exposure variables^a^\Percentile^b^	Min	5th	25th	50th	75th	95th	Max
As metabolites (μg g^−1^ crea)							
MMA	0.06	0.08	0.19	0.40 (1.10)^c^	1.67	6.14	28.5
DMA	0.07	3.09	11.27	20.73 (14.58)	29.5	70.75	129.1
iAs	0.11	0.19	0.41	0.83 (0.61)	1.33	4.74	6.55
tAs	0.34	3.76	12.09	23.19 (16.29)	33.29	76.02	137.5

^a^Abbreviations: iAs represents the sum of As^3+^ and As^5+^; MMA: methylarsonic acid; DMA: dimethylarsinic acid; tAs: the sum of iAs, MMA, and DMA; μg g^−1^ crea: μg per g creatinine

^b^Pregnant women from Maternal Infant cohort in Taiwan (*n* = 64). Limit of detection for As^3+^ is 0.09 μg/L, As^5+^ is 0.05 μg/L, for MMA it is 0.05 μg/L and for DMA it is 0.04 μg/L

^c^The values inside parenthesis are the average value of unadjusted arsenic expressed as μg/L

**Fig. 1:**
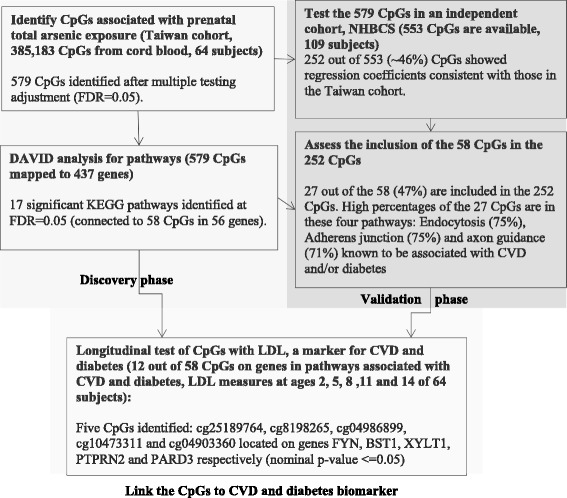
The flow of analyses performed in the study

**Fig. 2:**
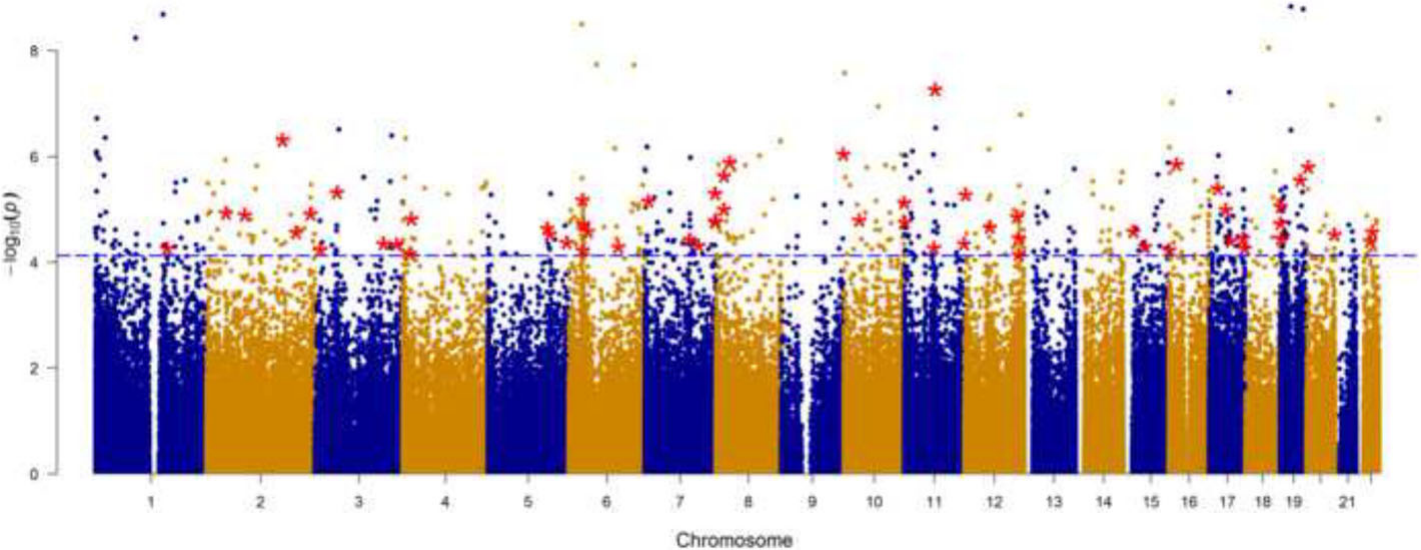
Manhattan plot for Genome-wide DNA methylation associated with creatinine adjusted urinary arsenic concentration. The horizontal dashed blue line corresponds to the significance threshold *p* = 7.51E-05 (FDR Adjusted *p*-value <= 0.05), red color stars represent the CpG sites corresponding to genes enriched in KEGG pathways from DAVID analysis (see Additional file 7: Table S3). Blue and golden colors are used to differentiate the chromosomes

After pre-processing the DNA methylation data (see the Methods section, and Additional file 1: Figure S1), 385,183 CpG sites were analyzed. The flow for the analyses is depicted in Fig. 1. Epigenome-wide assessments of statistical associations between log_10_ creatinine-adjusted maternal urinary arsenic level and logit transformed DNA methylation (also noted as M values) were conducted via robust regressions. Covariates included in robust regressions were child’s sex, batch of DNA methylation analyses, mother’s age, mother’s pre-pregnancy BMI, mother’s education level, and estimated proportions of six blood cell-types (Additional file 5: Table S1, related methods are in the Methods section). Figure 2 shows the Manhattan plot of *p*-values for testing on the 385,183 CpG sites, with a dashed blue line indicating the *p*-value threshold corresponding to FDR of *p* = 0.05 [31]. In total, 579 CpG sites showed statistically significant associations at FDR of 0.05. Additional file 6: Table S2 lists these 579 CpG sites along with their regression coefficients, *p*-values, and corresponding chromosomes, locations on the chromosomes, corresponding genes, and location on the genes. About 60% of these 579 CpGs showed a positive association between DNA methylation and in utero tAs. The majority of the CpG sites located in the North shore regions of the CpG Island had higher DNA methylation associated with higher in utero tAs and about 39% of these CpG sites were located upstream of transcription start site (TSS1500, TSS200) or 1st Exon (Additional file 6: Table S2).

**Fig. 3:**
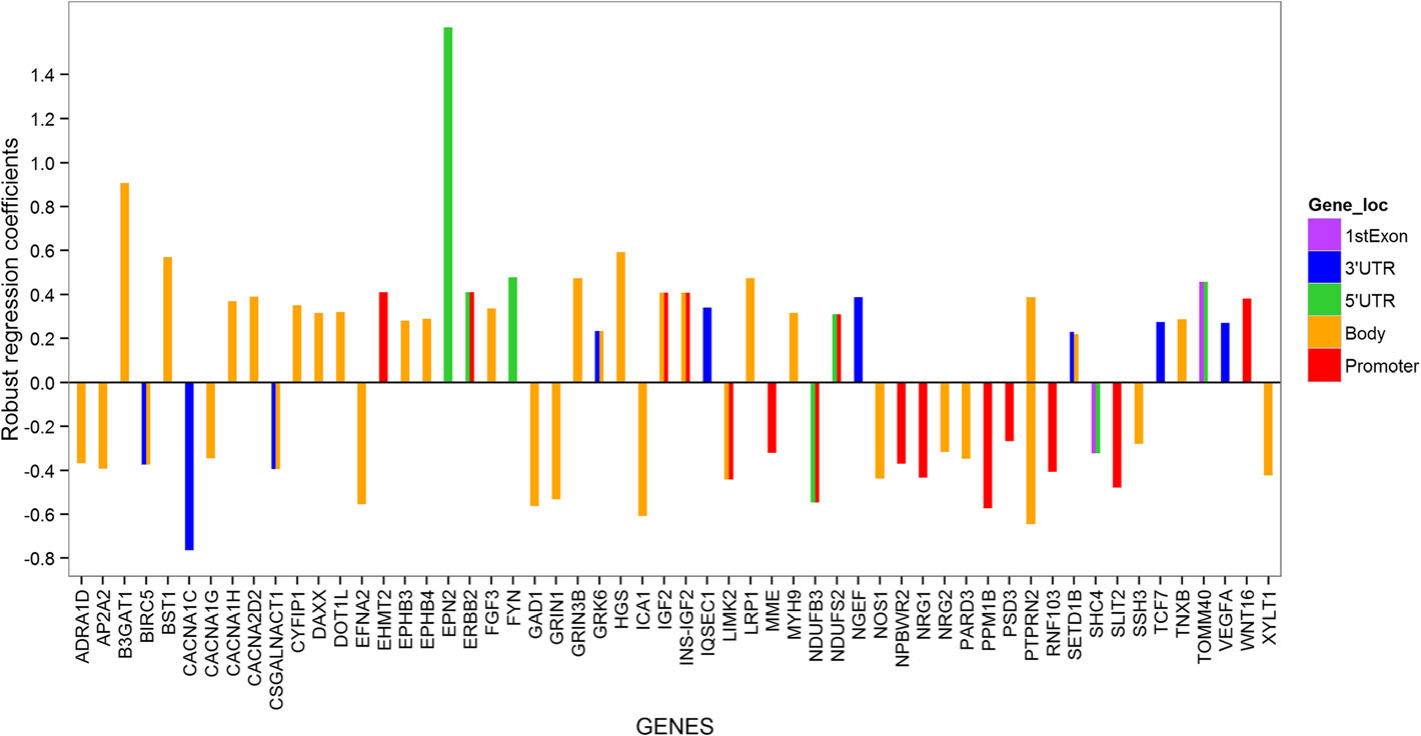
Association of arsenic exposure with the DNA methylation based on M-values of the 58 CpG sites mapped to 56 genes. The x-axis has the 56 genes enriched in KEGG pathways at FDR level of *p* = 0.05, while the y-axis shows the estimates of total arsenic coefficients related to 58 CpG sites from robust regression. Adjusting factors include cell counts, child’s sex, batch effect, mother’s age, mother’s BMI and mother’s education level. M-values are defined as log2 [β/(1-β)]. Different colors indicate the location of the CpGs on a gene

The 579 CpG sites were mapped to 437 genes (Additional file 6: Table S2), which were further analyzed using the bioinformatics tool DAVID [32, 33]. This analysis led to 17 significantly enriched KEGG pathways (at FDR = 0.05) and 58 CpGs were within the genes involved in these pathways (Additional file 7: Table S3), including pathways connected to CVDs and diabetes [34] (e.g., Type I and Type II diabetes mellitus, focal adhesion, calcium signaling pathway, adherens junction, and chondroitin sulfate biosynthesis [35]), pathways linked to neurological and cognitive abilities (Alzheimer’s disease and amyotrophic lateral sclerosis [ALS]), and pathways in cancer (the 58 CpG sites involved in these pathways are marked by red stars in Fig. 2). Among these 58 CpG sites corresponding to the genes enriched in KEGG pathways, most of them are located in the body region of a gene (Fig. 3). Majority of these 58 CpGs are located in the island region (~57%) or north shore (~22%). Furthermore, in approximately 55% out of the 58 CpG sites, we found that higher in utero tAs were linked to higher DNA methylation in cord blood, as indicated by positive regression coefficients in Fig. 3. The strongest association between in utero tAs and cord blood DNA methylation occurred at CpG cg23767840, which is in the 5’UTR region of gene *EPN2* (coding for the Epsin-2 protein).

The resulting 579 CpG sites from our study were further tested in the independent New Hampshire Birth Cohort Study (NHBCS) (*n* = 109). Details of the NHBCS cohort and findings of the replication study are included in the supplemental material (Additional file 3: Material 2). Of the 579 CpG sites 553 were available for analyses in NHBCS. We applied robust regression models with covariates comparable to those included in our study to assess the association of tAs with cord blood DNA methylation at these 553 CpG sites. At 46% of the 553 CpG sites (252 CpGs), the associations of in utero tAs with cord blood DNA methylation levels were consistent with those found in our study in terms of direction of regression coefficients, although none survived multiple testing. The 252 CpGs were mapped to 191 genes. Functional annotation analysis using DAVID on 191 genes identified following pathways (*p*-value < 0.05, although not surviving multiple testing via controlling of FDR): axon guidance, endocytosis, focal adhesion, adherens junction and cytokine-cytokine receptor interaction. Four of these five pathways were included in the 17 pathways identified in our cohort. In total, 12 CpGs in these pathways were in the 58 CpGs noted above.

**Fig. 4:**
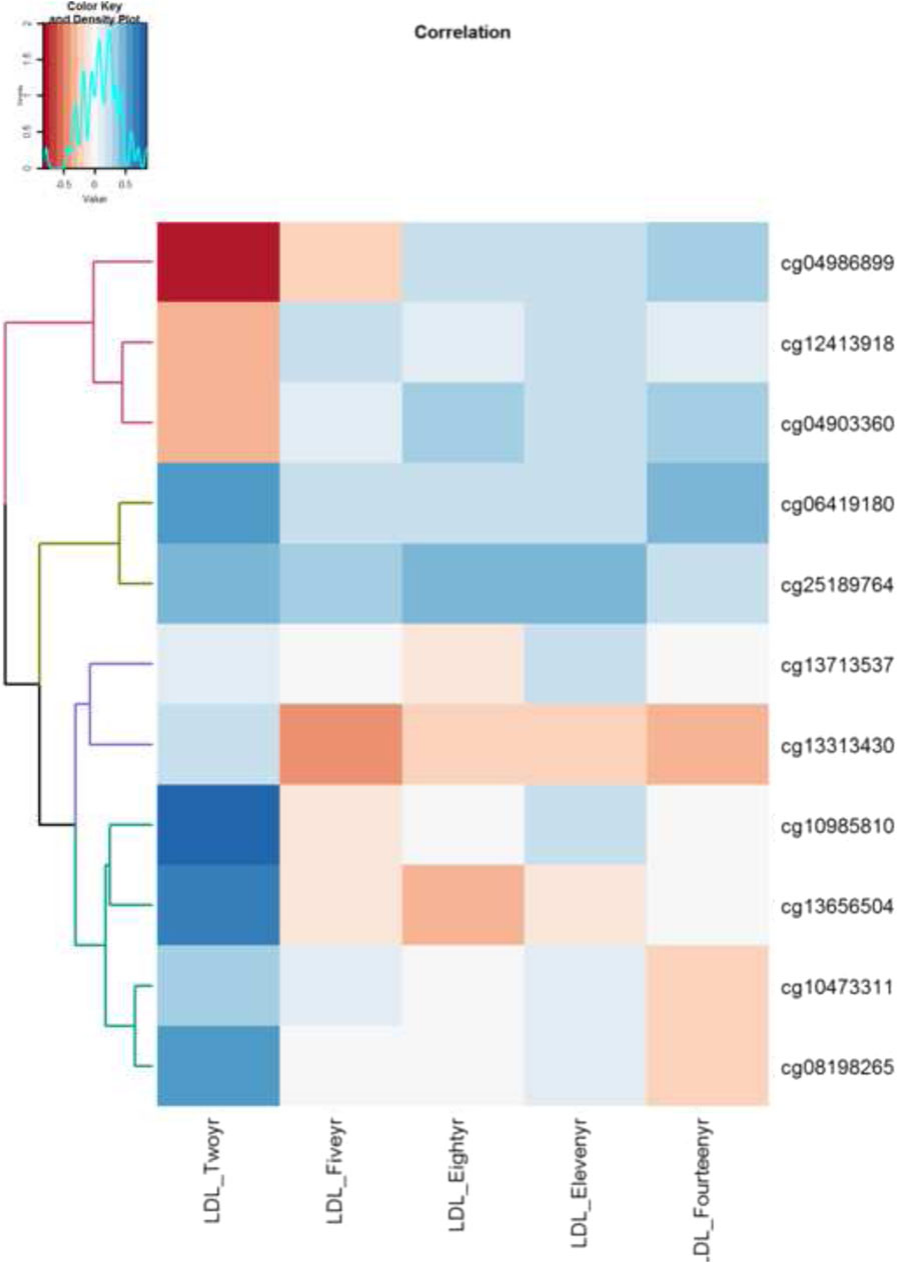
Heatmap of the correlations between cord blood DNA methylation and LDL across different ages (2, 5, 8, 11, 14 years)

In addition, 27 of the 252 CpGs are in the list of 58 CpGs (27/58 = ~47%) noted earlier (Additional file 7: Table S3). Genes corresponding to these 27 CpGs are more often linked to pathways involved in endocytosis, adherens junction, axon guidance (a neural developmental process in which neurons send out axons to reach the correct targets) and chondroitin sulfate biosynthesis. From linear mixed models, we found that in utero arsenic exposure was significantly associated with LDL (coeff = 0.17, *p*-value =0.04), after adjusting for the effects of covariates time, gender and birth weight. Given this observation and the connection of arsenic exposure with CVDs and diabetes noted in the literature [7, 8, 36, 37], findings from the pathway analyses, and findings in the replication study, we further investigated the CpG sites of the genes enriched in KEGG pathways that are potentially linked to cardiovascular diseases and diabetes in our Taiwan cohort. In particular, 11 CpGs (located on 10 genes, Additional file 6: Table S2) were included in this analysis and these 11 CpGs were among the 27 CpGs replicated in the NHBCS cohort. We assessed the association of cord blood DNA methylation at these CpGs with a biomarker of CVDs and diabetes, plasma low density lipoprotein (LDL). LDL was measured at multiple ages of the children (at 2, 5, 8, 11, and 14 years). Plasma LDL concentration is the most stable in humans, with or without fasting, among blood lipids such as triglycerides. Among the 11 CpGs, cord blood DNA methylation at some CpGs showed a pattern of positive correlations with LDL at each age. While some were negatively correlated with LDL at age 2 and positively correlated at later ages (Fig. 4), for most CpGs, the strongest correlations (positive or negative) occurred at age 2. In particular, the heatmap (Fig. 4) indicated that DNA methylation levels at two CpGs, cg06419180 and cg25189764, were positively correlated with the LDL at different ages, while the directions of correlations at the rest of the CpG sites seemed to change over time. Via linear mixed models, we tested the association of LDL with DNA methylation (with LDL at ages 2, 5, 8, 11 and 14 as the outcome, cell type compositions and batch-effect adjusted DNA methylation as the predictor, and child’s age, sex of the child, and birth weight as covariates) as well as the interaction effect between DNA methylation and age. We found that CpG cg25189764 had a statistically significant association with LDL (coefficient = 0.157, *p*-value = 0.047). DNA methylation at another 4 CpG sites showed statistically significant interaction with time (Table 4). After adding BMI Z-Score into the model, the main effect of cg25189764 was no longer statistically significant. However, the statistical significance of the interaction effects with time for the other four CpG sites was kept, and the estimates of the coefficients and *p*-values were minutely affected.

**Table 4:** Coefficients of the covariates included in linear mixed model for the five significant CpG sites

	Estimated Coefficients; *p*-value				
Variable	*cg25189764*	*cg08198265*	*cg04986899*	*cg10473311*	*cg04903360*
CpG	0.16; 0.04	0.498; 0.02	−0.362; 0.03	0.0145; 0.12	−0.189; 0.18
Age	0.017; <0.001	0.020; <0.001	0.015; <0.001	0.017; <0.001	0.016; <0.001
CpG*Age	--	−0.063; 0.00	0.030; 0.02	−0.021; 0.03	0.026; 0.03
Gender	0.022; 0.62	0.038; 0.40	0.037; 0.42	0.045; 0.35	0.021; 0.67
Birth Weight	−5.42 × 10^−6^; 0.93	4.38 × 10^−6^; 0.95	0.000044; 0.57	2.26 × 10^−6^; 0.97	2.01 × 10^−6^; 0.98

It is worth noting that DNA methylation at these 5 CpG sites was likely to be stable across the life course, based on findings in the Accessible Resource for Integrated Epigenomic Studies (ARIES) explorer[38]. The stability was evaluated via median and variances of DNAm over time using Gene view in ARIES explorer (http://www.ariesepigenomics.org.uk/ariesexplorer).

## Discussion

In utero arsenic exposure has been known to be associated with long term adverse health outcomes. Arsenic is also known to modify DNA methylation by inducing either global hypo-methylation [39, 40] or hyper-methylation [41]. The epigenetic marking acquired at earlier age has been known to be associated with phenotypic consequences later in life [42, 43]. This adverse health outcome can be due to the epigenetic modification caused by the in utero arsenic exposure. Thus the overall aim of this study was to identify CpG sites from cord blood that would represent biomarkers of possible adverse effects of in utero arsenic exposure in newborns and of future health outcomes. In total, at 579 CpGs identified from a cohort in Taiwan DNA methylation was associated with in utero arsenic exposure. To further understand the biological mechanisms of genes linked to these 579 CpG sites, a gene annotation analysis using DAVID was performed, which led to an identification of 17 statistically significant KEGG pathways. Genes corresponding to the identified CpGs are known to be involved in arsenic-associated diseases including neuronal [44–46], immune [47], cancer [48], cardiovascular and diabetes [8, 36, 37]. Experimental models have demonstrated a role of in utero acquired somatic epigenetic alternations in diseases [49–51]. Given the regulatory functionality of DNA methylation on different genes, the identified CpG sites may serve as epigenetic biomarkers of potential harmful effects of in-utero arsenic exposure among newborns.

Findings at 46% of the identified 579 CpG sites were replicated in an independent cohort, the NHBCS, with respect to directions of associations, though these did not survive multiple testing adjustments. However, the median tAs (without creatinine adjustment) in NHBCS was 2.8 μg/L with interquartile range (IQR) of 3.64 μg/L, which is substantially lower than that in the Taiwanese cohort (median = 11.51 μg/L and IQR = 16.80 μg/L). This difference, small sample sizes from both studies, differences in ancestry and unmeasured confounding may explain the limited agreement in the findings between the two cohorts.

The post hoc analysis on CpG sites replicated in the NHBCS cohort and related to genes enriched in KEGG pathways for cardiovascular disease and diabetes led to the identification of five CpG sites cg25189764, cg08198265, cg04986899, cg10473311 and cg04903360 located on genes *FYN, BST1, XYLT1, PTPRN2 and PARD3,* respectively. *FYN* is an important regulator of whole body metabolism and is known to be associated with insulin sensitivity in mice [52]. *BST-1* is a glycosyl-phosphatidylinositol (GPI) and is expressed in abundant in pancreatic islet cells [53]. Proteins containing a *GPI* anchor play key roles in a wide variety of biological processes [54]. *XYLT1* is involved in heparan sulfate (a type of glycosaminoglycan; GAG) biosynthesis [55, 56]. *GAG*s have been studied for their role as a potential target in treating CVDs [57, 58]. Protein encoded by *PTPRN2* (also known as *IAR*) is a known autoantigen in insulin-dependent diabetes mellitus [59]. *PARD3* has been identified as candidate gene for its association with type 2 diabetes in Mexican study [60]. Out of these five CpGs, cg25189764 is located in the 5’UTR of gene *FYN*, and the other four CpGs were located in the body of the genes. We observed that most CpG sites on genes enriched in KEGG pathways were located in the body region of a gene (Fig. 3). The regulatory functionality of DNA methylation on genes at those CpG sites is likely to be different from the functionality at CpG sites in the promoter region [61, 62]. Methylation in immediate vicinity of transcription start site (TSS; part of the promoter region) is known to block the transcription of gene, while methylation in the body region of gene might stimulate or act as markers of transcription [63, 64]. Further assessment on their associations with gene expressions will improve our understanding of their regulatory functionality.

The temporal stability in DNA methylation at the five CpG sites (cg25189764, cg08198265, cg04986899, cg10473311 and cg04903360) showing associations with LDL across different ages raised a possibility of long term consequences of DNA methylation, established in utero, on LDL at later life. More interestingly, for the four CpGs (cg08198265, cg04986899, cg10473311 and cg04903360), the DNA methylation effects were likely to change with age. Specifically, for cg08198265 and cg10473311, the effect of DNA methylation was positive before age 8 years, but negative after age 8 (this was obtained by plugging in age in years into the inferred models given in Table 4), and for cg04986899 and cg04903360, the association changed from negative to positive at ages 14 years and 8 years, respectively. Our analyses did not show that BMI Z-score is a potential confounder for the interaction effect of DNA methylation with time on LDL. Of interest, ages 11 and 14 are during adolescence, a period of significant changes, e.g., puberty, rapid growth, and often BMI increase.

A previous study in utero arsenic exposure in the NHBCS was reported by Koestler et al. [16]. The top 100 CpGs identified in Koestler et al. did not overlap with the 579 CpGs, although 25% of their 100 CpGs showed statistical significance at the 0.05 level in our study (not surviving multiple testing). The disagreement could have been driven by some key differences in the analytical methods. Koestler et al. categorized arsenic exposure levels into quartiles and applied analysis of covariance with tests for trends, while our study applied robust regressions to log10-transformed arsenic concentrations to take into account possible outliers. By categorizing a continuous variable, statistical testing power for testing the associations might have been reduced. In addition, Koestler et al. did not adjust for maternal BMI, nor the cell type proportions estimated using the *minfi* R package [20, 21], though they did explore associations between urinary arsenic and estimated cell-type proportions in cord blood.

We also compared the findings from our study with another epigenome-wide study by Broberg et al. [18]. The focus of that study also concentrated on the top CpG sites ranked by statistical significance on their association with in utero arsenic exposure, although none of the top CpG sites survived multiple testing corrections. The top CpG sites determined by Broberg et al. did not overlap with those identified in our study, nor overlapped with the top CpGs in Koestler et al. [16]. Broberg et al. [18] utilized linear regression and did not adjust for cell type heterogeneity. In addition, some top CpG sites discussed in Broberg et al. included annotated probe-SNPs (single nucleotide polymorphisms) located within 10 base-pairs of the target CpG. They can result in biased methylation measurements, and were excluded from our analysis. The study by Rojas et al. [17] identified 4771 CpG sites significantly associated with maternal urinary total arsenic. Among the 579 CpGs identified in our study from the cohort in Taiwan, 15 CpGs were present in the list of 4771 CpG sites. In addition, at these 15 CpGs, directions of associations (i.e., direction of coefficients) are consistent with those in Rojas et al. findings (see Additional file 8: Table S4).

It is worth noting that the four studies we discussed herein (Koestler et al. [16], Broberg et al. [18], Rojas et al. [17], and ours) were conducted in different regions (United States, Bangladesh, Mexico, and Taiwan, respectively) with vastly different medians in utero arsenic exposures which may have limited replicability (for tAs, in Koestler et al., median = 4.1 μg/L, in Broberg et al., median = 66 μg/L, in Rojas et al., median = 23.3 μg/L [65], and in our study, median = 11.51 μg/L (without creatinine adjustment)). It is also possible that ancestry, race/ethnicity or other regional differences may have contributed to the disagreement in the findings. In addition, all studies had small sample sizes (less than 200), so some of the findings are also likely to be false-positives. A large-scale study incorporating different races/ethnicities, with a wide exposure range, is well deserved. Our study had a benefit of replicating results using standard statistical approaches. Nonetheless, replicating DNA methylation analyses in additional populations, harmonizing, and comparing different DNA methylation studies on in utero arsenic exposure will help to assess the generalizability of the results. Future studies also should be directed at examining whether arsenic-related health outcomes are associated with cord blood DNA methylation in a long-term follow-up of the children in multiple cohorts.

## Conclusion

We found that in utero arsenic exposure was associated with cord blood DNA methylation. The genes corresponding to the identified CpG sites were involved in various pathways including signaling pathways, Type I and Type II diabetes mellitus, and neuroactive ligand-receptor interactions. Cord blood DNA methylation at cg25189764, cg08198265, cg04986899, cg10473311 and cg04903360 were associated with low-density lipoprotein (LDL) at later life. These CpGs need to be studied further for their role in cardiovascular disease and diabetes in arsenic-exposed populations*.* Although larger studies are needed, results from this study contributes to a better understanding of epigenetic mechanism of diseases related to in utero arsenic exposure in infants.

## Additional files

Additional file 1: Figure S1. Flow of data collection. (PDF 91 kb)

Additional file 2: Material 1 Data Collection, Pre-processing, and Cell Mixture Assessment . (DOCX 23 kb)

Additional file 3: Material 2 Description of NHBCS. (DOCX 26 kb)

Additional file 4: Figure S2. Histogram of Total Urinary arsenic concentration. (PNG 92 kb)

Additional file 5: Table S1. Cell proportions for 6 cell types. (XLSX 14 kb)

Additional file 6: Table S2. CpG sites identified from Taiwanese study and replicated in NHBCS. (XLSX 70 kb)

Additional file 7: Table S3. Genes and KEGG pathways corresponding to 58 CpG sites. (DOCX 14 kb)

Additional file 8: Table S4. CpG sites consistent between Taiwanese and Rojas et al. study. (XLSX 12 kb)

## Abbreviations

coeff: Coefficient coeff.m coefficient for main effect coeff.int Coefficient for interaction effect
CpG: 5′-cytosine-phosphate-guanine-3′; CpGs: Multiple CpG
DAVID: Database for Annotation, Visualization and Integrated Discovery
DNA: Deoxyribonucleic acid
DNAm: DNA methylation
FDR: False discovery rate
KEGG: Kyoto Encyclopedia of Genes and Genomes
LDL: Low density lipoprotein
NHBCS: New Hampshire Birth Cohort Study
tAs: Total arsenic obtained by adding inorganic arsenic (iAs), mono-methylated arsenic (MMA), di-methyl arsenic (DMA)
TSS: Transcription start site; TSS1500: within 1500 base pairs of a TSS; TSS200: within 200 base pairs of TSS.

## References

[1] Nordstrom DK (2002). Public health. Worldwide occurrences of arsenic in ground water. Science.

[2] Guan H, Piao F, Zhang X, Li X, Li Q, Xu L (2012). Prenatal exposure to arsenic and its effects on fetal development in the general population of Dalian. Biol Trace Elem Res.

[3] Smith AH, Marshall G, Liaw J, Yuan Y, Ferreccio C, Steinmaus C (2012). Mortality in young adults following in utero and childhood exposure to arsenic in drinking water. Environ Health Perspect.

[4] Chou WC, Chung YT, Chen HY, Wang CJ, Ying TH, Chuang CY (2014). Maternal arsenic exposure and DNA damage biomarkers, and the associations with birth outcomes in a general population from Taiwan. PLoS One.

[5] Rosenberg HG (1974). Systemic arterial disease and chronic arsenicism in infants. Arch Pathol.

[6] Hawkesworth S, Wagatsuma Y, Kippler M, Fulford AJ, Arifeen SE, Persson LA (2013). Early exposure to toxic metals has a limited effect on blood pressure or kidney function in later childhood, rural Bangladesh. Int J Epidemiol.

[7] Yuan Y, Marshall G, Ferreccio C, Steinmaus C, Selvin S, Liaw J (2007). Acute myocardial infarction mortality in comparison with lung and bladder cancer mortality in arsenic-exposed region II of Chile from 1950 to 2000. Am J Epidemiol.

[8] Davila-Esqueda ME, Morales JM, Jimenez-Capdeville ME, De la Cruz E, Falcon-Escobedo R, Chi-Ahumada E (2011). Low-level subchronic arsenic exposure from prenatal developmental stages to adult life results in an impaired glucose homeostasis. Exp Clin Endocrinol Diabetes.

[9] Rossman TG, Klein CB (2011). Genetic and epigenetic effects of environmental arsenicals. Metallomics.

[10] Gluckman PD (2012). Epigenetics and metabolism in 2011: Epigenetics, the life-course and metabolic disease. Nat Rev Endocrinol.

[11] Vickers MH (2014). Early life nutrition, epigenetics and programming of later life disease. Nutrients.

[12] O'Sullivan L, Combes AN, Moritz KM (2012). Epigenetics and developmental programming of adult onset diseases. Pediatr Nephrol.

[13] Majumdar S, Chanda S, Ganguli B, Mazumder DN, Lahiri S, Dasgupta UB (2010). Arsenic exposure induces genomic hypermethylation. Environ Toxicol.

[14] Smeester L, Rager JE, Bailey KA, Guan X, Smith N, Garcia-Vargas G (2011). Epigenetic changes in individuals with arsenicosis. Chem Res Toxicol.

[15] Xie Y, Liu J, Benbrahim-Tallaa L, Ward JM, Logsdon D, Diwan BA (2007). Aberrant DNA methylation and gene expression in livers of newborn mice transplacentally exposed to a hepatocarcinogenic dose of inorganic arsenic. Toxicology.

[16] Koestler DC, Avissar-Whiting M, Houseman EA, Karagas MR, Marsit CJ (2013). Differential DNA methylation in umbilical cord blood of infants exposed to low levels of arsenic in utero. Environ Health Perspect.

[17] Rojas D, Rager JE, Smeester L, Bailey KA, Drobna Z, Rubio-Andrade M (2015). Prenatal arsenic exposure and the epigenome: identifying sites of 5-methylcytosine alterations that predict functional changes in gene expression in newborn cord blood and subsequent birth outcomes. Toxicol Sci.

[18] Broberg K, Ahmed S, Engstrom K, Hossain MB, Jurkovic Mlakar S, Bottai M (2014). Arsenic exposure in early pregnancy alters genome-wide DNA methylation in cord blood, particularly in boys. J Dev Orig Health Dis.

[19] Kile ML, Houseman EA, Baccarelli AA, Quamruzzaman Q, Rahman M, Mostofa G (2014). Effect of prenatal arsenic exposure on DNA methylation and leukocyte subpopulations in cord blood. Epigenetics.

[20] Jaffe AE, Irizarry RA (2014). Accounting for cellular heterogeneity is critical in epigenome-wide association studies. Genome Biol.

[21] Houseman EA, Accomando WP, Koestler DC, Christensen BC, Marsit CJ, Nelson HH (2012). DNA methylation arrays as surrogate measures of cell mixture distribution. BMC Bioinformatics.

[22] Gilbert-Diamond D, Cottingham KL, Gruber JF, Punshon T, Sayarath V, Gandolfi AJ (2011). Rice consumption contributes to arsenic exposure in US women. Proc Natl Acad Sci U S A.

[23] Maksimovic J, Gordon L, Oshlack A (2012). SWAN: subset-quantile within array normalization for illumina infinium HumanMethylation450 BeadChips. Genome Biol.

[24] Aryee MJ, Jaffe AE, Corrada-Bravo H, Ladd-Acosta C, Feinberg AP, Hansen KD (2014). Minfi: a flexible and comprehensive Bioconductor package for the analysis of Infinium DNA methylation microarrays. Bioinformatics.

[25] Smyth GK. Limma: linear models for microarray data. Bioinformatics and Computational Biology Solutions Using R and Bioconductor. Edited by: Gentleman R, Carey V, Dudoit S, R Irizarry WH. New York: Springer; 2005. p. 397-420.

[26] Smyth GK, Yang YH, Speed T (2003). Statistical issues in cDNA microarray data analysis. Methods Mol Biol.

[27] Shirasawa T, Ochiai H, Ohtsu T, Nishimura R, Morimoto A, Hoshino H (2013). LDL-cholesterol and body mass index among Japanese schoolchildren: a population-based cross-sectional study. Lipids Health Dis..

[28] Huang DW, Sherman BT, Tan Q, Collins JR, Alvord WG, Roayaei J (2007). The DAVID Gene functional classification tool: a novel biological module-centric algorithm to functionally analyze large gene lists. Genome Biol.

[29] Fraser A, Macdonald-Wallis C, Tilling K, Boyd A, Golding J, Davey Smith G (2013). Cohort Profile: the Avon longitudinal study of parents and Children: ALSPAC mothers cohort. Int J Epidemiol.

[30] Boyd A, Golding J, Macleod J, Lawlor DA, Fraser A, Henderson J (2013). Cohort Profile: the 'children of the 90s'--the index offspring of the Avon longitudinal study of parents and Children. Int J Epidemiol.

[31] Benjamini Y, Hochberg Y. Controlling the false discovery rate: a practical and powerful approach to multiple testing. J R Stat Soc Ser B Methodol. 1995:289–300.

[32] Huang da W, Sherman BT, Lempicki RA (2009). Systematic and integrative analysis of large gene lists using DAVID bioinformatics resources. Nat Protoc.

[33] Huang da W, Sherman BT, Lempicki RA (2009). Bioinformatics enrichment tools: paths toward the comprehensive functional analysis of large gene lists. Nucleic Acids Res.

[34] Chan KH, Huang YT, Meng Q, Wu C, Reiner A, Sobel EM (2014). Shared molecular pathways and gene networks for cardiovascular disease and type 2 diabetes mellitus in women across diverse ethnicities. Circ Cardiovasc Genet.

[35] Gowd V, Gurukar A, Chilkunda ND (2016). Glycosaminoglycan remodeling during diabetes and the role of dietary factors in their modulation. World J Diabetes.

[36] Wang SL, Chiou JM, Chen CJ, Tseng CH, Chou WL, Wang CC (2003). Prevalence of non-insulin-dependent diabetes mellitus and related vascular diseases in southwestern arseniasis-endemic and nonendemic areas in Taiwan. Environ Health Perspect.

[37] Gribble MO, Howard BV, Umans JG, Shara NM, Francesconi KA, Goessler W (2012). Arsenic exposure, diabetes prevalence, and diabetes control in the strong heart study. Am J Epidemiol.

[38] Relton CL, Gaunt T, McArdle W, Ho K, Duggirala A, Shihab H (2015). Data resource Profile: accessible resource for integrated Epigenomic studies (ARIES). Int J Epidemiol.

[39] Reichard JF, Schnekenburger M, Puga A (2007). Long term low-dose arsenic exposure induces loss of DNA methylation. Biochem Biophys Res Commun.

[40] Coppin JF, Qu W, Waalkes MP (2008). Interplay between cellular methyl metabolism and adaptive efflux during oncogenic transformation from chronic arsenic exposure in human cells. J Biol Chem.

[41] Mass MJ, Wang L (1997). Arsenic alters cytosine methylation patterns of the promoter of the tumor suppressor gene p53 in human lung cells: a model for a mechanism of carcinogenesis. Mutat Res.

[42] Relton CL, Groom A, St Pourcain B, Sayers AE, Swan DC, Embleton ND (2012). DNA methylation patterns in cord blood DNA and body size in childhood. PLoS One.

[43] Godfrey KM, Sheppard A, Gluckman PD, Lillycrop KA, Burdge GC, McLean C (2011). Epigenetic gene promoter methylation at birth is associated with child's later adiposity. Diabetes.

[44] Luo J, Shu W. Arsenic-induced developmental neurotoxicity. Handbook Arsenic Toxicol. 2014;363

[45] Gong G, O'Bryant SE (2010). The arsenic exposure hypothesis for Alzheimer disease. Alzheimer Dis Assoc Disord.

[46] Vahidnia A, Romijn F, van der Voet GB, de Wolff FA (2008). Arsenic-induced neurotoxicity in relation to toxicokinetics: effects on sciatic nerve proteins. Chem Biol Interact.

[47] Lemarie A, Morzadec C, Bourdonnay E, Fardel O, Vernhet L (2006). Human macrophages constitute targets for immunotoxic inorganic arsenic. J Immunol.

[48] Hsu WL, Tsai MH, Lin MW, Chiu YC, Lu JH, Chang CH (2012). Differential effects of arsenic on calcium signaling in primary keratinocytes and malignant (HSC-1) cells. Cell Calcium.

[49] Perera F, Herbstman J (2011). Prenatal environmental exposures, epigenetics, and disease. Reprod Toxicol.

[50] Skinner MK (2011). Role of epigenetics in developmental biology and transgenerational inheritance. Birth Defects Res C Embryo Today.

[51] Skinner MK (2011). Environmental epigenetic transgenerational inheritance and somatic epigenetic mitotic stability. Epigenetics.

[52] Lee TW, Kwon H, Zong H, Yamada E, Vatish M, Pessin JE (2013). Fyn deficiency promotes a preferential increase in subcutaneous adipose tissue mass and decreased visceral adipose tissue inflammation. Diabetes.

[53] Kajimoto Y, Miyagawa J, Ishihara K, Okuyama Y, Fujitani Y, Itoh M (1996). Pancreatic islet cells express BST-1, a CD38-like surface molecule having ADP-ribosyl cyclase activity. Biochem Biophys Res Commun.

[54] Paulick MG, Bertozzi CR (2008). The glycosylphosphatidylinositol anchor: a complex membrane-anchoring structure for proteins. Biochemistry.

[55] Pedersen LC, Tsuchida K, Kitagawa H, Sugahara K, Darden TA, Negishi M (2000). Heparan/chondroitin sulfate biosynthesis. Structure and mechanism of human glucuronyltransferase I. J Biol Chem.

[56] Kreuger J, Kjellen L (2012). Heparan sulfate biosynthesis: regulation and variability. J Histochem Cytochem.

[57] Grande-Allen KJ, Osman N, Ballinger ML, Dadlani H, Marasco S, Little PJ (2007). Glycosaminoglycan synthesis and structure as targets for the prevention of calcific aortic valve disease. Cardiovasc Res.

[58] Ballinger ML, Nigro J, Frontanilla KV, Dart AM, Little PJ (2004). Regulation of glycosaminoglycan structure and atherogenesis. Cell Mol Life Sci.

[59] Schmidli RS, Colman PG, Cui L, Yu WP, Kewming K, Jankulovski C (1998). Antibodies to the protein tyrosine phosphatases IAR and IA-2 are associated with progression to insulin-dependent diabetes (IDDM) in first-degree relatives at-risk for IDDM. Autoimmunity.

[60] Below JE, Gamazon ER, Morrison JV, Konkashbaev A, Pluzhnikov A, McKeigue PM (2011). Genome-wide association and meta-analysis in populations from Starr County, Texas, and Mexico City identify type 2 diabetes susceptibility loci and enrichment for expression quantitative trait loci in top signals. Diabetologia.

[61] Weber M, Hellmann I, Stadler MB, Ramos L, Paabo S, Rebhan M (2007). Distribution, silencing potential and evolutionary impact of promoter DNA methylation in the human genome. Nat Genet.

[62] Pai AA, Bell JT, Marioni JC, Pritchard JK, Gilad Y (2011). A genome-wide study of DNA methylation patterns and gene expression levels in multiple human and chimpanzee tissues. PLoS Genet.

[63] Jones PA (2012). Functions of DNA methylation: islands, start sites, gene bodies and beyond. Nat Rev Genet.

[64] Moore LD, Le T, Fan G (2013). DNA methylation and its basic function. Neuropsychopharmacology.

[65] Laine JE, Bailey KA, Rubio-Andrade M, Olshan AF, Smeester L, Drobna Z (2015). Maternal arsenic exposure, arsenic methylation efficiency, and birth outcomes in the biomarkers of exposure to ARsenic (BEAR) pregnancy cohort in Mexico. Environ Health Perspect.

