# Supplementary material for: Genome-wide DNA methylation at birth in relation to in utero arsenic exposure and the associated health in later life
Source: Environ Health. 2017 May 30;16:50. doi: 10.1186/s12940-017-0262-0 (PMC5450181; doi:10.1186/s12940-017-0262-0)
Supplement: Supplementary file 1 — Flow of data collection. (PDF 91 kb) [file 12940_2017_262_MOESM1_ESM.pdf]

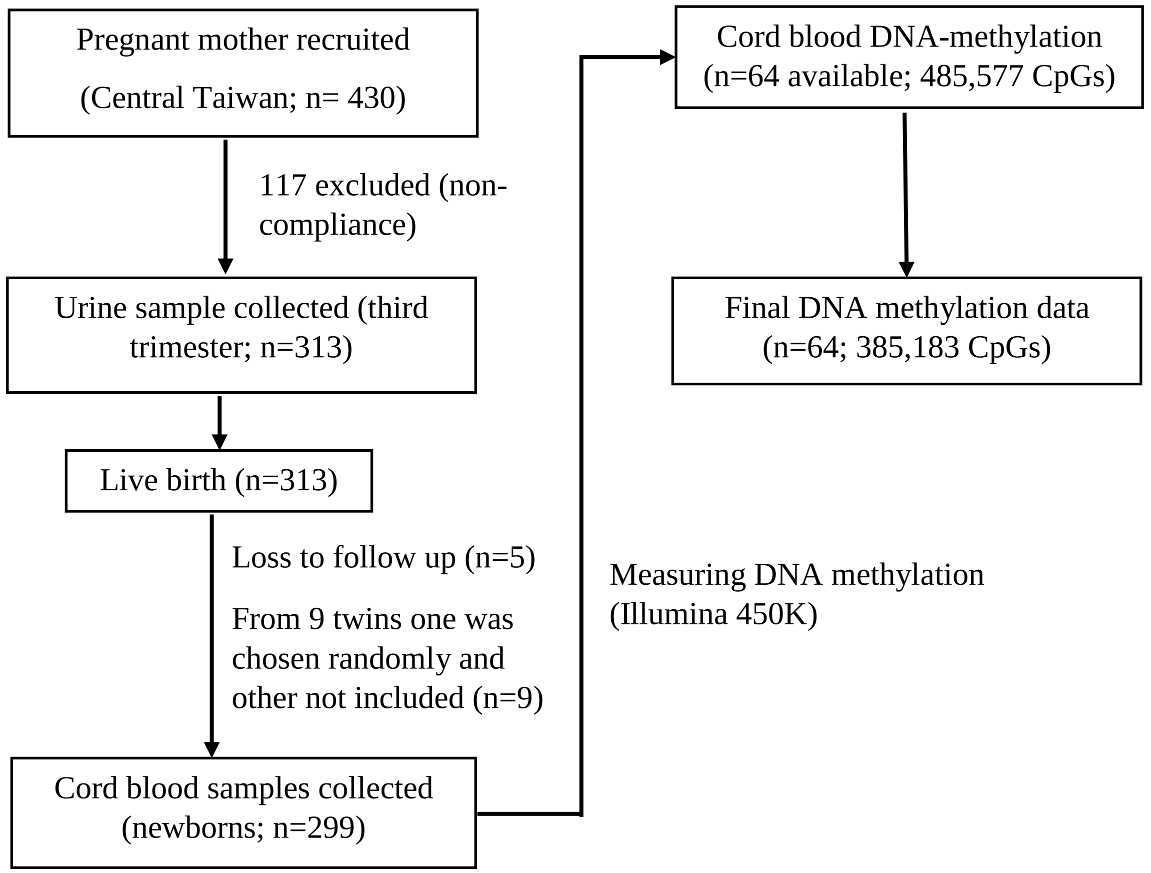

Figure S1. Subject recruitment and preprocessing of DNA methylation data in Taiwanese birth cohort
