# Supplementary material for: Genome-wide DNA methylation at birth in relation to in utero arsenic exposure and the associated health in later life
Source: Environ Health. 2017 May 30;16:50. doi: 10.1186/s12940-017-0262-0 (PMC5450181; doi:10.1186/s12940-017-0262-0)
Supplement: Supplementary file 2 — Material 1 Data Collection, Pre-processing, and Cell Mixture Assessment . (DOCX 23 kb) [file 12940_2017_262_MOESM2_ESM.docx]

**Assessment of arsenic exposure**

Participants provided a spot urine sample at the time of enrollment in this study (at eight weeks of gestation). Urine was frozen at -20 °C in a 10-ml polypropylene tube. Arsenite (As^III^), arsenate (As^V^), monomethylarsonic acid (MMA), and dimethylarsinic acid (DMA) were quantified by high-performance liquid chromatography/inductively coupled plasma mass spectrometry (HPLC-ICP-MS) and anion exchange columns (Hamilton PRP X-100 [10 mm particle size, 250 mm64.1 mm]). Total urinary arsenic (TUA) was calculated by adding iAs (As^III^ + As^V^) + MMA + DMA. The limitations of detection (LOD) for the various species were 0.09 mg/L for As^III^, 0.05 mg/L for As^V^, 0.05 mg/L for MMA, and 0.04 mg/L for DMA. Creatinine was measured by the Beckman Synchron LX20 auto-system (Beckman Coulter, Brea, CA, USA) in the central lab of Chung-Ho Memorial Hospital of Kaohsiung Medical University using a spectrophotometric method with picric acid as the reactive at 520 nm. We used total arsenic (tAs) as the sum of inorganic arsenic (As^III^ + As^V^) and organic arsenic (MMA and DMA) divided by urinary creatinine; this ratio was used in the subsequent analyses.

**DNA methylation**

DNA was isolated from cord blood samples using buffy coat isolated from EDTA-treated blood (Gentra Puregene; Qiagen, Hilden, Germany) and bisulfite converted using the EZ DNA Methylation kit. Samples were randomized across several plates for epigenome-wide DNA methylation assessment using the Illumina Infinium Human Methylation 450 BeadChip which simultaneously profiles the methylation status of > 485,000 CpG sites with single-nucleotide resolution across the human genome.

DNA methylation is measured using beta values, calculated as M/ (M+U+ε), where M is the methylation signal of the target CpG, U is the unmethylated signal and ε=100, is a constant to protect division by zero. Thus, average beta-value (β) represents the percent methylation of the target CpG site and its value ranges between 0 and 1.

**Quality control**

The raw data of DNA methylation were pre-processed to achieve high quality for data analyses. The function *PreprocessSWAN* in the Bioconductor package *minfi* was used for normalization, background correction and peak correction. The function *preprocessSWAN* uses subset within array normalization (SWAN) technique, which normalizes Infinium type I and type II probes together within a single array. This technique reduces technical variability between arrays by accounting for differences in the comparison of the two probe types between arrays [1]. CpG sites located on sex chromosome and annotated probe SNPs within 10bp of the target CpGs were dropped to eliminate bias caused by subject’s gender and bias due to genetic variability.

**Correction for cell mixture proportion**

Blood is a mixture of functionally and developmentally distinct cell populations [2]. Adjusting for this cell type will remove potential confounding effects of cell heterogeneity in DNA methylation in blood samples [3]. Cell type composition of the blood sample was calculated using function e*stimateCellCounts* in the R package *minfi* [4, 5]. IDAT files from 450k Illumina DNA methylation were used to estimate the proportion of 6 cell types: CD8T, CD4T, NK, Monocyte, Granulocyte and B-cell. Cell type proportions are provided in supplemental Table S3.

**DAVID**

Illumina Infinium 450K Human Methylation Beadchip array version 1.2 was used to map the significant CpG sites to USCS reference genome and identify genes associated with these CpG sites. Functional enrichment and pathway analysis of resulting genes was carried out using DAVID gene functional classification tool [6]. DAVID is a large gene-centered knowledgebase which integrates the diverse annotation resources in a centralized location. DAVID knowledgebase is based upon single-linkage algorithm called the DAVID Gene Concept, and it serves as a gene/protein IDs database. The DAVID gene functional classification tool aggregates a list of genes or associated biological terms into organized classes of related genes or biology. For a given gene list, DAVID identifies enriched functional-related gene groups and provides a visualization of genes on pathway maps such as KEGG [7]. KEGG pathway maps are collection of manually drawn pathways based upon knowledge gained from experiments on functions of the cell and its metabolism. Genetic interaction in KEGG pathway represents the network of molecular interaction and reactions of gene products.

References:

1. Maksimovic J, Gordon L, Oshlack A: **SWAN: Subset-quantile within array normalization for illumina infinium HumanMethylation450 BeadChips**. *Genome biology* 2012, **13**(6):R44.

2. Reinius LE, Acevedo N, Joerink M, Pershagen G, Dahlen SE, Greco D, Soderhall C, Scheynius A, Kere J: **Differential DNA methylation in purified human blood cells: implications for cell lineage and studies on disease susceptibility**. *PloS one* 2012, **7**(7):e41361.

3. Koestler DC, Christensen B, Karagas MR, Marsit CJ, Langevin SM, Kelsey KT, Wiencke JK, Houseman EA: **Blood-based profiles of DNA methylation predict the underlying distribution of cell types: a validation analysis**. *Epigenetics* 2013, **8**(8):816-826.

4. Jaffe AE, Irizarry RA: **Accounting for cellular heterogeneity is critical in epigenome-wide association studies**. *Genome biology* 2014, **15**(2):R31.

5. Houseman EA, Accomando WP, Koestler DC, Christensen BC, Marsit CJ, Nelson HH, Wiencke JK, Kelsey KT: **DNA methylation arrays as surrogate measures of cell mixture distribution**. *BMC bioinformatics* 2012, **13**:86.

6. Huang DW, Sherman BT, Tan Q, Collins JR, Alvord WG, Roayaei J, Stephens R, Baseler MW, Lane HC, Lempicki RA: **The DAVID Gene Functional Classification Tool: a novel biological module-centric algorithm to functionally analyze large gene lists**. *Genome biology* 2007, **8**(9):R183.

7. Kanehisa M, Goto S: **KEGG: kyoto encyclopedia of genes and genomes**. *Nucleic acids research* 2000, **28**(1):27-30.
