# Supplementary material for: Genome-wide DNA methylation at birth in relation to in utero arsenic exposure and the associated health in later life
Source: Environ Health. 2017 May 30;16:50. doi: 10.1186/s12940-017-0262-0 (PMC5450181; doi:10.1186/s12940-017-0262-0)
Supplement: Supplementary file 3 — Material 2 Description of NHBCS. (DOCX 26 kb) [file 12940_2017_262_MOESM3_ESM.docx]

**NHBCS Cohort Description:**

The New Hampshire Birth Cohort Study (NHBCS) is an ongoing prospective study that began in 2009 and includes over 1500 women from two regions of New Hampshire, USA, enrolled between 24-28 weeks gestation. Mothers were recruited into the cohort if they were literate in English, mentally competent, between 18–45 years old, and reported using a private, unregulated well as the primary source of home drinking water. Infants included in the cohort were singleton pregnancies. Pre- and post-delivery questionnaires were administered to collect self-reported sociodemographic, lifestyle, and medical history data, and a structured medical records review was employed to collect information from the pregnancy and delivery. Cord blood samples are collected on >80% of all deliveries. This study consisted of the first participants born in the study with available cord blood samples for DNA methylation analysis and mothers that were not missing for urinary arsenic or any of the covariate data (n=109).

**Arsenic in maternal urine:**

Measures of maternal urinary arsenic have been described thoroughly elsewhere [1]. Briefly, spot urine samples were collected between 24–28 weeks gestation with 30 μL of 10 mM diammonium diethyldithiocarbamate, then samples were frozen at –80°C until analysis. High-performance liquid chromatography inductively coupled plasma mass spectrometry (ICP-MS) system measured individual arsenic species. Samples with values below the limit of detection (LOD) were assigned a value equal to the LOD divided by the square root of two [2]. Total maternal urinary arsenic concentrations (U-As) were calculated as the sum of inorganic arsenic (As^III^ & As^V^), monomethylarsonic acid (MMA^V^) and dimethylarsinic acid (DMA^V^), which was then log_10_-transformed prior to analyses.

**Cord blood DNA-M processing and QA/QC:**

DNA was bisulfite converted using the EZ DNA Methylation kit and subsequently subjected to epigenome-wide DNA methylation assessment using the Illumina Infinium® HumanMethylation450 BeadChip (Illumina, San Diego, CA) at the University of Minnesota Genomics Core Facility (Minneapolis, MN) following standardized protocols. Post-array processing was conducted in the ‘minfi’ package in R. Samples in which >2% of probes had poor detection p-values were excluded; probes with detection p-values > 0.01 in at least one sample were also removed. Functional normalization (funNorm) and ComBat were utilized to remove technical variations in the data; removal of batch effects was confirmed with principal components analysis. The normalized and batch-corrected beta-values were then transformed into M-values via log_2_(β/(1-β)) prior to statistical analyses.

**Covariates:**

Maternal age, maternal BMI, and estimated cell proportions were included as continuous covariates. Child gender and mother’s education were included as dichotomous covariates. Maternal education was defined as those with at least a college degree vs. those without a college degree. Cell type proportions were estimated with the current gold standard method [3] via the minfi package in R. In regression models, 5 of the 6 cell types were included as covariates (NK cells were excluded) to account for overall cellular heterogeneity.

**Replication Sample**

In total, 109 cord blood samples for which DNA-M had been obtained and complete arsenic and covariate data were available. Of the 579 CpG sites identified in the cohort in Taiwan, 553 were available for replication analyses within the NHBCS.

**Predictor and Covariate Distributions within NHBCS:**

| **Categorical** | **Frequency** | **%** |
| --- | --- | --- |
| Male Infant | 55 | 50.5 |
| Lower Maternal Education | 37 | 33.9 |
| **Continuous** | **Mean** | **S.D.** |
| Maternal Total Urinary Arsenic^*^ | 2.80 | 3.64 |
| Maternal Age at Enrollment | 31.02 | 4.39 |
| Pre-Pregnancy Maternal BMI | 24.24 | 4.20 |
| **Cell Proportions** | **Mean** | **S.D.** |
| CD4^+^ T-Cells | 0.12 | 0.04 |
| CD8^+^ T-Cells | 0.12 | 0.03 |
| NK Cells | 0.02 | 0.03 |
| B-Cells | 0.10 | 0.03 |
| Monocytes | 0.10 | 0.02 |
| Granulocytes | 0.58 | 0.07 |

^*^ Presenting the median and IQR due to skewed distribution.

**Statistical Analyses:**

For all 553 regressions, we tested the linear relationship between maternal total urinary As and cord blood DNA-M M-values while adjusting for confounders. Since batch effects were removed via ComBat during data processing, no batch variable was included in these analyses. The following model, consistent with the model used in the study based on data from Taiwan, was fit using robust regression via the lmFit function in the limma package in R (version 3.2.2), confidence intervals were extracted using the confint=TRUE option.

M-values = log_10_(U-As) + Child Gender + Urine Creatinine + Mother’s Age + Mother’s BMI + Mother’s Education + Estimated Cell Proportions

**Results:**

Arsenic regression coefficients, confidence intervals, and p-values (raw and FDR-adjusted p-values) are in Table S2 (the columns labeled as “NHBCS”).

**References:**

[1] D. Gilbert-Diamond, K.L. Cottingham, J.F. Gruber, T. Punshon, V. Sayarath, A.J. Gandolfi, et al., Rice consumption contributes to arsenic exposure in US women, Proc. Natl. Acad. Sci. 108 (2011) 20656–20660. doi:10.1073/pnas.1109127108.

[2] D.R. Helsel, Less than obvious: Statistical treatment of data below the detection limit, Environ. Sci. Technol. 24 (1990) 1766–1774.

[3] E.A. Houseman, W.P. Accomando, D.C. Koestler, B.C. Christensen, C.J. Marsit, H.H. Nelson, et al., DNA methylation arrays as surrogate measures of cell mixture distribution, BMC Bioinformatics. 13 (2012).
