# Supplementary figures and images for: Genome-wide DNA methylation at birth in relation to in utero arsenic exposure and the associated health in later life
Source: Environ Health. 2017 May 30;16:50. doi: 10.1186/s12940-017-0262-0 (PMC5450181; doi:10.1186/s12940-017-0262-0)

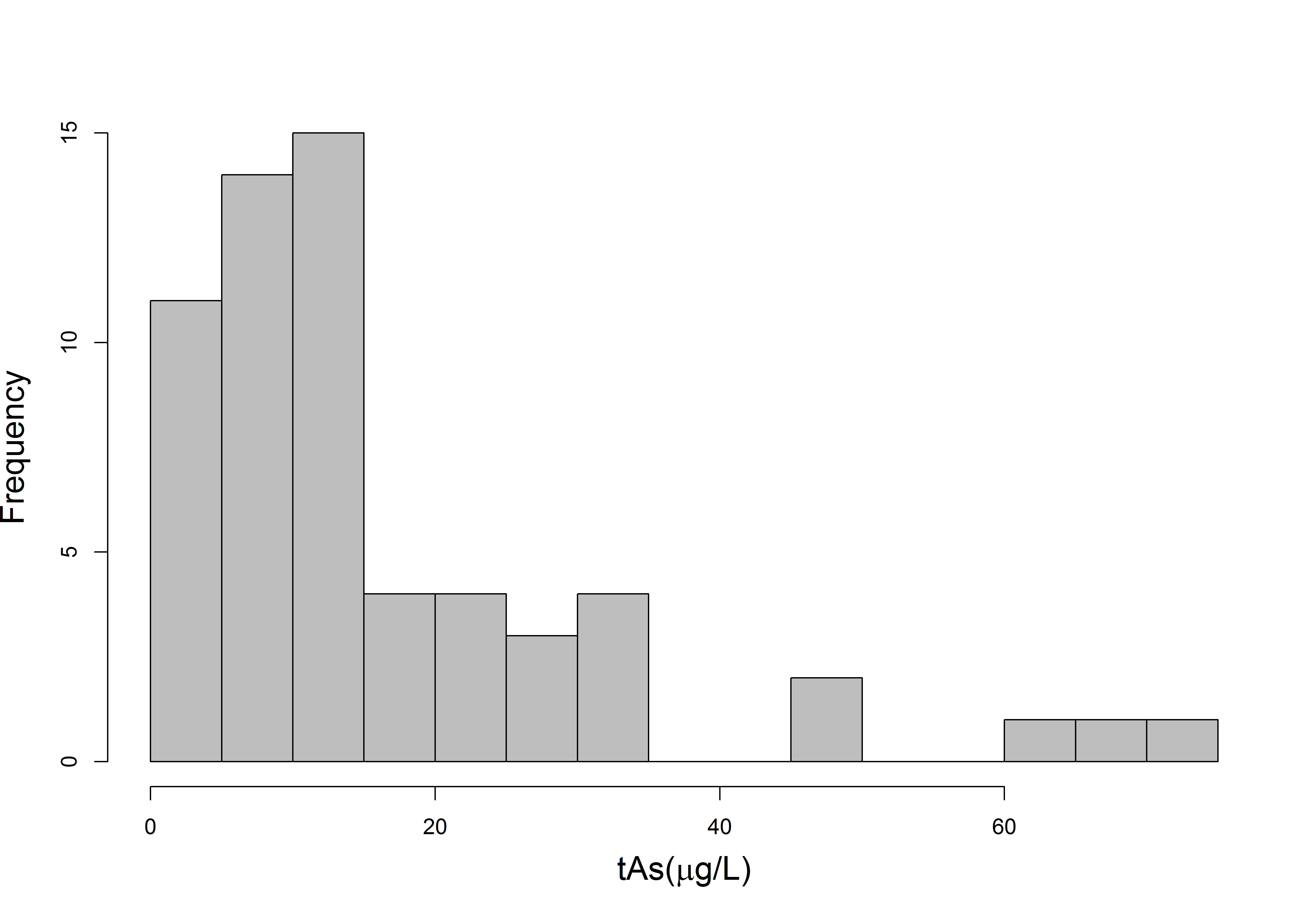

Supplement: Supplementary file 4 — Histogram of Total Urinary arsenic concentration. (PNG 92 kb) [file 12940_2017_262_MOESM4_ESM.png]
