# Supplementary material for: Genome-wide DNA methylation at birth in relation to in utero arsenic exposure and the associated health in later life
Source: Environ Health. 2017 May 30;16:50. doi: 10.1186/s12940-017-0262-0 (PMC5450181; doi:10.1186/s12940-017-0262-0)
Supplement: Supplementary file 7 — Genes and KEGG pathways corresponding to 58 CpG sites. (DOCX 14 kb) [file 12940_2017_262_MOESM7_ESM.docx]

**Table S3**. KEGG pathways identified using DAVID that are more specific to arsenic exposure based on data from n=64 pregnant women from the maternal infant cohort in Taiwan.

| **KEGG-Pathways** | **Genes** | **Adjusted p-value (FDR=0.05)** |
| --- | --- | --- |
| Calcium signaling pathway | NOS1*, BST1*, ERBB2*, GRIN1, CACNA1G, CACNA1H, CACNA1C, ADRA1D | 0.000011 |
| Endocytosis | PARD3*, AP2A2*, RNF103, PSD3, GRK6*, HGS*, IQSEC1*, EPN2 | 0.0000010 |
| Axon guidance | NGEF*, LIMK2, FYN*, EFNA2*, EPHB3*, EPHB4, SLIT2* | 0.000015 |
| Alzheimer's disease | NDUFB3, NOS1*, LRP1, GRIN1, MME, CACNA1C, NDUFS2* | 0.000033 |
| MAPK signaling pathway | CACNA1G, CACNA1H, PPM1B*, CACNA1C, CACNA2D2, DAXX*, FGF3 | 0.00057 |
| Regulation of actin cytoskeleton | LIMK2, INS-IGF2, SSH3, CYFIP1*, IGF2, MYH9*, FGF3 | 0.0019 |
| Type I diabetes mellitus | ICA1*, INS-IGF2, PTPRN2*, IGF2, GAD1 | 0.0018 |
| Amyotrophic lateral sclerosis (ALS) | NOS1*, GRIN1, TOMM40, DAXX* | 0.0037 |
| Adherens junction | TCF7, PARD3*, FYN*, ERBB2* | 0.0087 |
| Pathways in cancer | WNT16*, TCF7, ERBB2*, VEGFA, BIRC5, FGF3 | 0.0091 |
| ErbB signaling pathway | ERBB2*, NRG1, NRG2, SHC4 | 0.01 |
| Focal adhesion | TNXB*, FYN*, ERBB2*, VEGFA, SHC4 | 0.0097 |
| Chondroitin sulfate biosynthesis | CSGALNACT1*, B3GAT1, XYLT1* | 0.01 |
| Neuroactive ligand-receptor interaction | PARD3*, GRIN1, NPBWR2, GRIN3B*, ADRA1D | 0.017 |
| Lysine degradation | DOT1L*, SETD1B, EHMT2* | 0.034 |
| Type II diabetes mellitus | INS-IGF2, CACNA1G, IGF2, CACNA1C | 0.038 |
| Huntington's disease | NDUFB3, AP2A2*, GRIN1, NDUFS2* | 0.045 |

* CpG sites of these genes were consistently associated (in terms of regression coefficient) with total urinary arsenic exposure in an independent cohort NHBCS.
